# Results of brexucabtagene-autoleucel for patients with relapsed/refractory Mantle Cell Lymphoma in the routine setting in Germany and Switzerland

**DOI:** 10.1038/s41409-025-02789-7

**Published:** 2026-01-16

**Authors:** Linda Simon, Vladan Vucinic, Kai Rejeski, Maria-Luisa Schubert, Enver Aydilek, Eva-Maria Wagner-Drouet, Olaf Penack, Malte von Bonin, Bastian von Tresckow, Marcel Teichert, Reinhard Marks, Christiane Pott, Martin Fehr, Corinna Leng, Roland Schroers, Christian Koenecke, Johannes Duell, Stephan Stilgenbauer, Fabian Mueller, Judith S. Hecker, Uta Brunnberg, Nicolaus Kroeger, Kai Kronfeld, Matthias Theobald, Anke Ohler, Irene Schmidtmann, Georg Hess, Peter Dreger

**Affiliations:** 1 Department of Hematology and Medical Oncology, University Medical School of the Johannes Gutenberg-University Mainz, Mainz, Germany; 2 Department of Hematology, Cellular Therapy, Hemostaseology and Infectious Diseases, University of Leipzig, Leipzig, Germany; 3 Department of Medicine III, Ludwig-Maximilian University Hospital, Munich, Germany; 4 Department of Hematology, Oncology and Rheumatology and German Working Group for Hematopoietic Stem Cell Transplantation and Cellular Therapy, Heidelberg University Hospital, Heidelberg, Germany; 5 Department for Hematology and Medical Oncology, University Medical Center Goettingen, Goettingen, Germany; 6 Department of Hematology, Oncology and Tumorimmunology, Charité University Hospital Berlin, Berlin, Germany; 7 Department of Internal Medicine I, University Hospital, TU Dresden, Dresden, Germany; 8 Department of Hematology and Stem Cell Transplantation, West German Cancer Center and German Cancer consortium (DKTK partner site Essen), University Hospital Essen, University of Duisburg-Essen, Essen, Germany; 9 Department of Hematology, Oncology and Stem Cell Transplantation, Faculty of Medicine, University of Freiburg, Freiburg, Germany; 10 Second Medical Department, University Hospital Schleswig-Holstein, Kiel, Germany; 11 Division of Oncology and Hematology, Cantonal Hospital St. Gallen, St. Gallen, Switzerland; 12 Swiss Cancer Institute Competence Center, Bern, Switzerland; 13 Med. Department of Hematology, Oncology and Cancer Immunology, Charité -University Hospital Berlin, Campus Benjamin Franklin, Berlin, Germany; 14 Knappschaftskrankenhaus Bochum, University Hospital Bochum, Bochum, Germany; 15 Department of Hematology, Hemostasis, Oncology, and Stem Cell Transplantation, Hannover Medical School, Hannover, Germany; 16 St. Bernward Krankenhaus, Hildesheim, Germany; 17 Department of Internal Medicine II, Hematology and Oncology, University Hospital Wuerzburg, Wuerzburg, Germany; 18 Department of Internal Medicine III, Ulm University, Ulm, Germany; 19 Department of Internal Medicine 5, Hematology and Oncology, Friedrich-Alexander University Erlangen-Nuernberg, Erlangen, Germany; 20 Department of Medicine III, School of Medicine and Health, TranslaTUM, Center for Translational Cancer Research, Technical University of Munich (TUM), Munich, Germany; 21 Department of Medicine, Hematology and Oncology, Goethe University Frankfurt, Frankfurt am Main, Germany; 22 Interdisciplinary Clinic for Stemcell Transplantation, Center for Oncology, University Hospital Hamburg Eppendorf, Hamburg, Germany; 23 Interdisciplinary Center for Clinical Trials Mainz, University Medical School of the Johannes Gutenberg-University Mainz, Mainz, Germany; 24 Institute for Medical Biostatistics, Epidemiology and Informatics, University Medical Center of the Johannes Gutenberg University Mainz, Mainz, Germany

**Keywords:** Drug development, Epidemiology

## Introduction

Mantle Cell Lymphoma (MCL) is an incurable B-cell neoplasm, representing 6–8% of newly diagnosed B-cell lymphomas [1]. Brexucabtagene-autoleucel (brexu-cel, Tecartus®) is a CD19-targeting chimeric antigen receptor (CAR) T-cell therapy approved in Europe for treatment of relapsed/refractory (r/r) MCL after two prior lines of therapy, including a Bruton’s tyrosine kinase inhibitor (BTKi) [2]. Approval was based on a small population of 68 patients in the ZUMA-2 trial [2]. Therefore, data on toxicities and long-term outcomes in a real-world setting are of great value to confirm the potential and show limitations of brexu-cel.

With this intent, we performed a retrospective real-world analysis of brexu-cel as standard-of-care (SOC) treatment for r/r MCL in Germany and Switzerland.

## Patients and methods

Data were extracted from the European Mantle Cell Lymphoma Registry (EMCL-R) and the German Registry for Stem cell Transplantation (Deutsches Register für Stammzelltransplantation und Zelltherapie, DRST). Written informed consent in accordance with the European data protection regulations and the Declaration of Helsinki was obtained.

Eligibility included r/r MCL, age ≥18 years and treatment prior to Oct. 2023 within the early access program or as SOC. Last follow-up was October 31st, 2024. Median follow-up for the entire population was 17 months.

All data underwent plausibility checks, source data review and a defined query process.

Overall survival (OS) was defined as time from brexu-cel infusion to death from any cause, event-free survival (EFS) as time from brexu-cel infusion to relapse or disease progression, the initiation of a new treatment or death from any cause, whichever occurred first. Probabilities of EFS and OS were evaluated by Kaplan–Meier estimates and log-rank tests. Difference was considered significant if a *p-*value < 0.05 was detected. The Holm-method was used for adjustment for multiplicity. Statistical analyses were performed in collaboration with the Institute for Medical Biostatistics, Epidemiology and Informatics (IMBEI, Mainz) using SAS Version 9.4 and GraphPad Prism Version 10.3.

## Results

113 patients were included from 18 German and one Swiss center. Baseline characteristics are displayed in S1 and 2. Median time from diagnosis to CAR-T therapy was 63 months, prior treatment lines included autologous and allogeneic hematopoietic stem cell transplantation (allo-HSCT) (63% and 10%, respectively). Seventy-three patients (65%) had been refractory to the last treatment prior to brexu-cel indication or experienced relapse within 12 months after initiation (POD12). Twenty-six patients (23%) did not fulfill the ZUMA-2 eligibility criteria due to history of allo-HSCT, ECOG > 1 or > 5 prior lines of therapy. *TP53* mutation was present in 27%, Ki-67 > 30% in 79% and blastoid variant in 33% of patients. Bridging was administered to 89% of patients (S3 and 4).

Out of 85 patients with response data available, 61% showed complete remission (CR), 27% partial remission (PR) and 12% stable/progressive disease (SD/PD). Median duration of response was 31 months (S5 and 6).

**Fig. 1:**
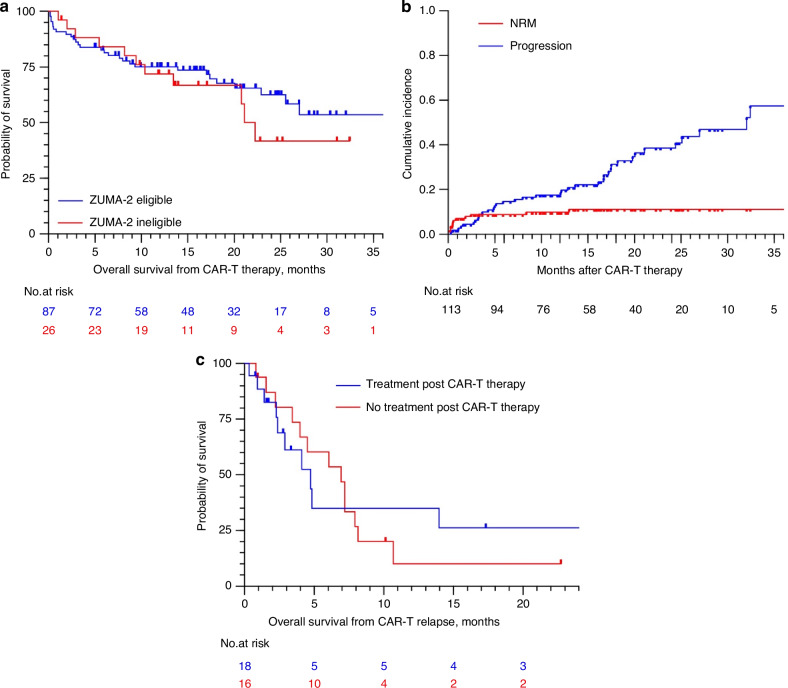
Response and survival after CAR-T therapy in r/r MCL. Kaplan–Meier plots of patients after CAR-T therapy. **a** Overall survival (OS) after CAR-T therapy for ZUMA-2 eligible vs. non-eligible patients, **b** cumulative incidence with competing risk non-relapse-mortality (NRM) and progression and **c** OS for patients with post-CAR**-**T progression/relapse and with or without treatment post-CAR-T therapy.

Median EFS and OS after CAR-T cell infusion for the total study population were 25 and 41 months, respectively (Fig. 1a).

Regarding risk factors, only the use of chemoimmunotherapy compared to BTKi was associated with inferior OS and EFS (*p* = 0.03 and *p* = 0.04, respectively, S7–10) in univariable analysis including adjustment for multiplicity. In multivariable analysis, only patients with POD12 showed poorer survival (*p* = 0.017 for EFS and *p* = 0.015 for OS, S11–13).

Among 92 patients with available information, 13% showed reconstitution of B-cells and 30% of T-cells within six months. B- or T-cell recovery was defined as detection of B-cells or CD4^+^-T-cell counts >200/µL [3]. Intravenous immunoglobulins (IVIG) were supplemented in 46 patients (42%). IVIG supplementation was associated with improved EFS and OS but lacked significant difference after exclusion of patients who died within the first 30 days after CAR-T treatment (S14 and 15).

After CAR-T therapy, 67 clinically significant infections occurred in 35 of 113 patients (31%), with bacterial pneumonia being the dominant cause (30%), followed by COVID-19-associated pneumonia (30%) (S14). Six infections (9%) caused NRM, comprising COVID-19-associated pneumonia (*n* = 1), bacterial pneumonia (*n* = 2), sepsis with macrophage activation syndrome and multi-organ failure (*n* = 1), neutropenic infection (*n* = 1) and meningitis (*n*= 1).

At data cutoff, 41 patients had died, 29 due to MCL and 12 without prior disease recurrence (S5 and 16). Whereas 8 of 12 non-relapse deaths occurred within one month post CAR-T therapy (early NRM, median 10 days, 6–32), the remaining 4 deaths were observed at a median duration of 188 days post brexu-cel (87-442, late NRM). Competing risk analysis showed an increased probability for NRM compared to progression during the first 4 months post CAR-T therapy and a plateau after 13 months, while the risk for progression increased steadily (Fig. 1b). For early NRM, CAR-T therapy-associated complications were the main cause, whereas infections were accounted primarily for late NRM, with at least two cases associated to long-term lymphopenia.

In total, 40 patients were either refractory to brexu-cel or relapsed/progressed after initial response, among which 18 received a follow-up treatment (S17). Median OS after CAR-T failure was 4.8 months (Fig. 1c).

## Discussion

The advent of brexu-cel has marked a change of paradigms for patients with r/r MCL after BTKi failure. However, recent data have demonstrated relapses and NRM events attributed to CAR-T-associated toxicities and infections [4, 5]. This registry study investigated the efficacy and safety of brexu-cel, focusing on NRM and the outcome after brexu-cel failure.

Although patients were older, more heavily pretreated and presented with more comorbidities as compared to the ZUMA-2 population [2], response rates and survival data was comparable to ZUMA-2 data and real-world analyses [6, 7]. Multivariable analysis revealed POD12 after last treatment as the only significant factor for poorer prognosis which aligns with previous trials [5, 6]. As already demonstrated by Yang et al. [8], our findings presume the existence of an additional adverse genetic profile (for example, mutations in TP53, SMARCA4, NOTCH2, KMT2D, and CDKN2A) in patients with POD12 and documented high-risk biology.

A major complication after CAR-T treatment in MCL were infections, which confirms previous findings [2, 4, 5]. Infections recorded ranged from viral respiratory infections to bacteriemia and opportunistic infections and lead to death in 27% of patients (compared to 9% in ZUMA) [2].

Our data suggests that prolonged suppression of B-cell and T-cell immunity is an important driver of infections. All patients with late NRM (*n* = 4) had reported B-cell-aplasia. The numeric survival benefit of patients that received immunoglobulins after CAR-T therapy indicates a potential benefit of IVIG supplementation (HR for OS 0.49).

NRM events occurred predominantly early and were mainly driven by typical cytokine-related CAR-T toxicities. The NRM one-year cumulative incidence of 13% was higher than values previously reported (8% [9] and 7.35% [2]). However, despite the risk for infections over the entire course post CAR-T, failure and disease progression remain the greatest challenge.

The outcome of patients who did not respond or relapsed after brexu-cel was very poor. Treatment after CAR-T failure achieved only short-termed responses (median OS 4.8 months). To this end, the introduction of non-covalent BTKi or bispecific antibodies in the MCL treatment landscape may be beneficial [10].

Limitations of this study are those inherent to retrospective registry analyses. Since data were collected before first introduction of ICAHT in 2023 [11], hematotoxicity and associated scores were not evaluated.

While brexu-cel offers new perspectives for patients with refractory MCL, NRM remains a concern in the first months post CAR-T therapy, with infections as main driver. Optimization of treatment sequence for patients with POD12 and the development of effective salvage therapies after failure of CAR-T-therapy will be crucial in the treatment of MCL.

## Supplementary information

Supplemental material

## Data Availability

The datasets generated and analyzed during this study can be made available upon reasonable request to the corresponding author. Decisions regarding data sharing will be made on a case-by-case basis considering data protection and other applicable regulations.
