# Supplementary material for: Results of brexucabtagene-autoleucel for patients with relapsed/refractory Mantle Cell Lymphoma in the routine setting in Germany and Switzerland
Source: Bone Marrow Transplant. 2026 Jan 16;61(4):480–3. doi: 10.1038/s41409-025-02789-7 (PMC13056568; doi:10.1038/s41409-025-02789-7)
Supplement: Supplementary file 1 — Supplemental material [file 41409_2025_2789_MOESM1_ESM.docx]

## Data Source

The EMCL-R is a European registry, currently involving 79 German and 13 Swiss hospitals. The registry focusses on capturing the entire disease course, with data being centrally reviewed. The DRST is a central organization of all German adult transplant centers and collects data on cellular therapies in cooperation with the European Society of Blood and Marrow Transplantation (EBMT). The study is part of the EMCL-R which was approved by the Ethical Committee of Rhineland-Palatinate (Ref. Nr. 2018-13856). This study was conducted under the auspices of the German Lymphoma Alliance (GLA), the EMCL-Network and the Swiss Cancer Institute Competence Center (SCI).

## Study Design

All centers were trained and qualified by the manufacturer of brexu-cel (Kite-Gilead) for CAR-T cell administration and management of toxicities. As per federal directive, all centers were required to adhere to defined quality assurance measures based on the guidelines of the German Board of Oncology/Hematology for management of CAR-T cell toxicities.

## Treatment and Clinical Assessment

Baseline clinical information, data on tumor biology, therapy lines before and after brexu-cel administration, safety and efficacy outcomes were collected. Bridging therapy was defined as any treatment intended to control lymphoma between brexu-cel indication and the start of lymphodepleting chemotherapy.

Therapy decisions including bridging therapies and post-CAR-T follow-up were at the discretion of the treating physicians. CRS and ICANS were graded according to American Society for Transplantation and Cellular Therapy criteria^1^. Clinically relevant infections required either the need for hospitalization or any antibiotic treatment. Management of CAR-T-associated complications was performed according to institutional standards. Lymphoma response was assessed by the treating physician according to 2014 Lugano criteria^2^. IVIG substitution followed institutional guidelines. Early NRM was defined as death due to causes unrelated to lymphoma relapse or progression within approximately one month following brexu-cel administration, whereas late NRM was defined as death occurring beyond one month after CAR-T administration^3^.

## Statistical Methods

Multivariable analysis was performed using Cox proportional hazards model and excluding patients with missing data. Cumulative incidence of NRM and risk of MCL relapse or progression were estimated using competing risk models. Robustness of the results was assessed by comparing ZUMA-2-eligible patients with those enrolled in the pivotal study. In case of unreported data, available case analysis was implemented for statistical evaluation.

## Limitations

Limitations include bias due to varying degrees of complete data collection and as-treated analysis. Due to the small number of events, multivariable analysis of NRM was not possible. No data on CAR-T cell expansion or secondary malignancies were obtained.

1. Lee DW, Santomasso BD, Locke FL, et al. ASTCT Consensus Grading for Cytokine Release Syndrome and Neurologic Toxicity Associated with Immune Effector Cells. *Biol Blood Marrow Transplant*. 2019;25(4):625-638. doi:10.1016/j.bbmt.2018.12.758

2. Cheson BD, Ansell S, Schwartz L, et al. Refinement of the Lugano Classification lymphoma response criteria in the era of immunomodulatory therapy. *Blood*. 2016;128(21):2489-2496. doi:10.1182/blood-2016-05-718528

3. Lemoine J, Bachy E, Cartron G, et al. Nonrelapse mortality after CAR T-cell therapy for large B-cell lymphoma: a LYSA study from the DESCAR-T registry. *Blood Adv*. 2023;7(21):6589-6598. doi:10.1182/bloodadvances.2023010624

**Table S1 – Patient and tumor characteristics at start of CAR-T therapy**

| (n=information available) |  | At CAR-T therapy |
| --- | --- | --- |
| Patient |  |  |
| Sex [n (%)]  (n=110) | Female  Male | 21 (19.1)  89 (80.9) |
| ECOG [n (%)]  (n=46) | 0-1  2-3 | 43 (93.5)  3 (6.5) |
| Age, years [Median (range)] |  | 67 (49-84) |
| Disease |  |  |
| *TP53* mutation prior CAR-T [n (%)] (n=67) | Yes  No | 18 (26.9)  49 (73.1) |
| Maximum Ki67 [n (%)] (n=67) | 0-10 %  10-30 %  ≥ 30% | 3 (4.5)  11 (16,4)  53 (79,1) |
| Maximum MIPI [n (%)], (n=81) | High  Intermediate  low | 40 (49.4)  23 (28.4)  18 (22.2) |
| Last histology [n (%)], (n=57) | Classical  CLL-like  Blastoid / Pleomorph | 37 (64.9)  1 (1.8)  19 (33.3) |
| Time from diagnosis to CAR-T, months [Median (range)] | All (n=107)  < 70 years at CAR-T therapy  ≥ 70 years at CAR-T therapy | 63.2 (5.1 – 225.4)  85.9 (5.1 – 225.4)  104.7 (16.1-149.4) |
| Prior therapy lines |  |  |
| Number of lines [Median (range)] |  | 3 (1-8) |
| Prior allogeneic HSCT  [n (%)] | Yes  No | 10 (8.8)  103 (91.2) |
| Prior BTKi^1^ [n (%)] | Yes  No | 113 (100)  0 (0) |
| Reason for end of BTKi [n (%)] | Progression  Intolerance/Toxicity  Patient/Physician choice  Other/unknown | 66 (58.4)  9 (8.0)  11 (9.7)  27 (23.9) |
| Bendamustine < 1 year prior CAR-T [n (%)] | Yes  No | 13 (13.5)  96 (86.5) |
| POD12 prior CAR-T therapy  [n (%)] | Yes  No | 73 (64.6)  40 (35.4) |
| ZUMA-2 eligible^2^ [n (%)] | Yes  No | 87 (77.0)  26 (23.0) |

**Supplement 1**: ^1^Ibrutinib n=110, Zanubrutinib n= 2, Acalabrutinib n=1, ^2^ECOG > 1 at CAR-T therapy, > 5 therapy lines, history of allogeneic stem cell transplantation

Abbreviations: BTKi = Bruton´s Tyrosine Kinase inhibitor, CAR-T= chimeric antigen receptor T-cell, CLL= Chronic Lymphatic Leukemia, ECOG= Eastern Cooperative Oncology Group, HSCT= hematopoetic stem cell transplantation MIPI=Mantle Cell International Prognostic Index, POD12 = Progression of disease within 12 months

**Table S2: Additional patient characteristics**

| (n=information available) |  | At CAR-T therapy |
| --- | --- | --- |
| Patient |  |  |
| Age, years [n (%)]  (n= 111 at CAR-T therapy) | < 70  ≥ 70 | 63 (56.8)  48 (43.2) |
| Number of comorbidities ^1^ (n=97) | 0-1  2-5 | 64 (66.0)  33 (34.0) |
| Disease |  |  |
| Stage at diagnosis (Lugano)  [n (%)], (n=93) | 1-2  3-4 | 8 (8.6)  85 (91.4) |
| CNS involvement, at any time  [n (%)], (n=56) | Yes  No | 3 (5.4)  53 (94.6) |
| Bulky disease (7.5cm)  (n= 58 at CAR-T therapy) | Yes  No | 10 (17.2)  48 (82.8) |
| Risk factors by age at CAR-T therapy |  |  |
| *TP53* mutation at any time [n (%)], (n=18)  *p*-value= 0.0047 | < 70 years  ≥ 70 years | 15 (83.3)  3 (16.7) |
| Maximum Ki67 ≥ 30% [n (%)], (n=51) | < 70 years  ≥ 70 years | 25 (49.0)  26 (51.0) |
| Blastoid morphology [n (%)], (n=27) | < 70 years  ≥ 70 years | 14 (51.9)  13 (48.1) |
| Time from diagnosis to CAR-T, months [Median (range)] | All (n=107)  < 70 years at CAR-T therapy  ≥ 70 years at CAR-T therapy | 63.2 (5.1 – 225.4)  85.9 (5.1 – 225.4)  104.7 (16.1-149.4) |
| Prior therapy lines |  |  |
| Prior CD20-antibody [n (%)] | Yes  No | 112 (99.1)  1 (0.9) |
| Prior autologous HSCT  [n (%)] | Yes  No | 63 (55.8)  50 (44.2) |

**Supplement 2**: ^1^arrhythmia, cardiac disease, diabetes, cerebrovascular disease, depression, HIV, renal disease, pulmonary disease, skin cancer

Abbreviations: CAR-T= chimeric antigen receptor T-cell, CNS=central nervous system, HSCT= hematopoetic stem cell transplantation MIPI=Mantle Cell International Prognostic Index

**Table S3: Bridging Strategies prior CAR-T therapy**

| Bridging Strategies |  | All patients |
| --- | --- | --- |
| Time between indication for CAR-T therapy (tumorboard decision) and CAR-T therapy, days [Median (range)] |  | 68.5 (0-365) |
| Bridging prior CAR-T therapy [n (%)] (n=113) | Yes  No  Unknown | 101 (89.4)  11 (9.7)  1 (0.9) |
| Bridging regimens  [n (%)] (n=101) | Chemoimmunotherapy  Venetoclax (mono or + CD20/RT)  BT (mono or + CD20/RT)  BTKi + Venetoclax (+ CD20)  Lenalidomide + CD20  Radiotherapy mono  Anti-CD20 mono | 35 (34.7)  10 (9.9)  28 (27.7)  11 (10.9)  11 (10.9)  5 (5.0)  1 (1.0) |
| Response to bridging [n (%)] (n=76)^2^ | CR  PR  SD  PD | 12 (15.8)  35 (46.1)  17 (22.4)  12 (15.9) |
| Disease status at lymphodepletion  [n (%)], n=90 | CR  PR  SD  PD/ active disease | 12 (13.3)  35 (38.9)  17 (18.9)  26 (28.9) |

**Supplement 3**: ^1^ for statistical analysis of influence of BTKi on survival, only patients with BTKi pause < 30d prior CAR-T therapy were evaluated, ^2^ unknown or not evaluated in n=14 and n=22

Abbreviations: BTKi = Bruton´s Tyrosine Kinase inhibitor, POD12 = Progression of disease within 12 months, n.e. = not evaluated

**Table S4: Bridging therapy administered to MCL patients before and after T-cell apheresis**

| Bridging therapy prior CAR-T therapy | N |
| --- | --- |
| Rituximab – Bendamustine | 6 |
| Rituximab – DHAP | 3 |
| DHAP + Ibrutinib + Venetoclax | 1 |
| R-DHOX | 2 |
| R-BAC | 4 |
| VRCAP | 2 |
| High-dose Methotrexate | 2 |
| MATRIX + Venetoclax + Ibrutinib | 1 |
| MTX + AraC | 1 |
| R-Cytarabine | 3 |
| Gemcitabine / Oxaliplatin | 3 |
| R-CHOP | 3 |
| R-CHP + Velcade | 1 |
| O-ICE | 1 |
| R-ICE + Venetoclax | 1 |
| HAD + Venetoclax | 1 |
| ViP | 1 |
| Venetoclax | 7 |
| Venetoclax + CD20-Antibody | 2 |
| Venetoclax + CD20-Antibody + Radiotherapy | 1 |
| Ibrutinib | 20 |
| Ibrutnib + CD20 Antibody | 2 |
| Acalabrutinib | 1 |
| Ibrutinib + intrathecal chemotherapy + Radiotherapy | 1 |
| Ibrutinib + intrathecal chemotherapy | 1 |
| Ibrutinib + Radiotherapy | 2 |
| Zanubrutinib + CD20 Antibody | 1 |
| Lenalidomid | 10 |
| Lenalidomid + CD20 Antibody + Ibrutinib | 1 |
| Rituximab | 1 |

**Supplement 4**: Bridging therapies administered as tumor control to MCL patients before and after T-cell apheresis

Abbreviations: DHAP= Dexamethasone, Cisplatin, Highdose-Cytarabine, R-DHOX= Rituximab-Dexamethasone, Highdose-Cytarabine, Oxaliplatin, R-BAC= Rituximab-Bendamustine, Cytarabine, VRCAP= Vincristin, Rituximab, Cyclophosphamide, Doxorubicin, Prednisone, MATRIX= Methotrexate, Cytarabine, Thiotepa, Rituximab, MTX= Methotrexate, AraC= Cytarabine, R-CHOP= Rituximab, Cyclophosphamide, Doxorubicine, Vincristin, Prednisone, R-CHP= Rituximab, Cyclophosphamide, Doxorubicine, Prednisone, O-ICE= Obinutizumab-Ifosfamid, Carboplatin, Etoposide, R-ICE= Rituximab-Ifosfamid, Oxaliplatin, Etoposide, HAD= Highdose-Cytarabine, Dexamethasone, ViP= Vincristin, Prednisone

**Table S5 – Response after CAR-T therapy**

| (n=information available) |  | All patients evaluable |
| --- | --- | --- |
| Remission after CAR-T therapy [n (%)], (n=85) | CR  PR  SD  PD | 52 (61.2)  23 (27.1)  5 (5.9)  5 (5.9) |
| Duration of response [median, months (range)] | All patients  Zuma-2 ineligible patients | 31.0 (0.3-40.5)  21.1 (2.0-32.5) |
| Event-free-survival [median, months (range)] | All patients  Zuma-2 ineligible patients | 24.7 (0.2-40.5)  21.1 (1.4-32.5) |
| Overall-survival [median, months (range)] | All patients  Zuma-2 ineligible patients | 40.5 (0.2-40.5)  22.3 (1.4-32.5) |
| End of Observation |  |  |
| Status at End of Observation [n (%)] (n=113) | Alive  Progression  Remission  Dead | 72 (63.7)  11 (15.3)  61 (84.7)  41 (36.6) |
| Dead at End of Observation [n (%)] (n=41) | Progression  Remission | 29 (70.7)  12 (29.3) |
| Reason of Death [n (%)] (n=41) | MCL related  Toxicity  Infection  Other^1^ | 25 (61.0)  4 (9.6)  11 (26.8)  1 (2.4) |
| Death within one month post-CAR-T therapy [n (%)] (n=41) | Yes  No | 8 (19.5)  33 (80.5) |

**Supplement 5**: ^1^unknown. Abbreviations: CR=complete remission, n.e. = not evaluated, n.r. = not reached, OS = overall survival, PD=progressive disease, PR=partial remission, SD=stable disease

**Figure S6: Response and survival after CAR-T therapy**

| A  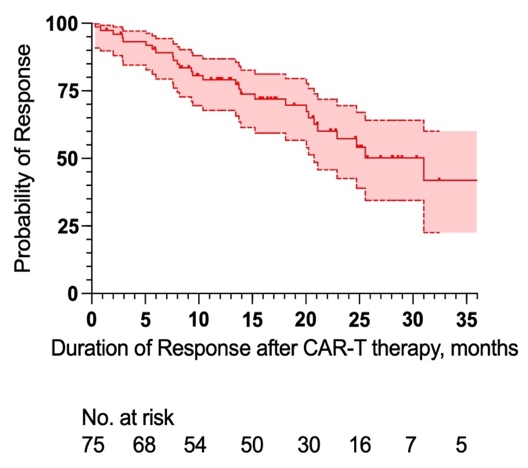 | B  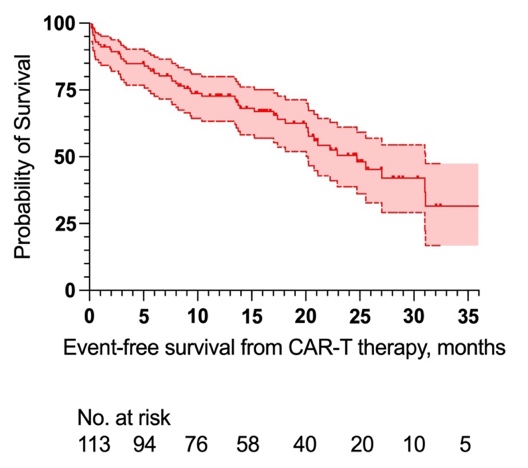 |
| --- | --- |
| C  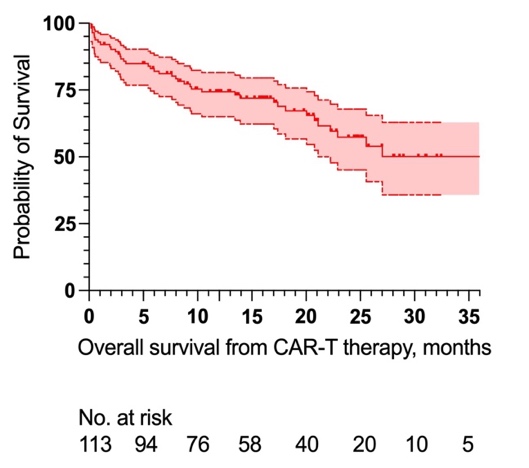 |  |

**Supplement 6**: Kaplan-Meier plots of patients after CAR-T therapy. (A) Duration of response (DoR) after CAR-T therapy with 95% CI for all patients, (B) event free survival (EFS) with 95% CI for all patients, (C) Overall survival (OS) with 95% CI for all patients

**Table S7: Univariable analysis of factors associated with outcome**

| Characteristics | N, % | OS (HR, 95 CI, p-value) | EFS (HR, 95 CI, p-value) |
| --- | --- | --- | --- |
| Sex |  |  |  |
| Female | 21 (19.1) | Reference | Reference |
| Male | 89 (80.9) | 0.83, 0.38 – 1.81, p= 0.62 | 0.86, 0.42 – 1.78, p= 0.67 |
| Age at CAR-T therapy |  |  |  |
| ≤70 | 63 (56.8) | Reference | Reference |
| >70 | 48 (43.2) | 1.31, 0.76 – 2.28, p= 0.32 | 1,31, 0.76 – 2.28, p= 0.32 |
| Number of comorbidities |  |  |  |
| ≤1 | 64 (66.0) | Reference | Reference |
| >1 | 33 (34.0) | 1.37, 0.66 – 2.83, p= 0.37 | 1.29, 0.69 – 2.46, p= 0.41 |
| Morphology |  |  |  |
| classical | 37 (64.9) | Reference | Reference |
| Blastoid/pleomorphic | 19 (33.3) | 1.75, 0.83 – 3.70, p= 0.17 | 1.58, 0.76 – 3.2, p= 0.20 |
| CLL-like | 1 (1.8) | Not evaluated | Not evaluated |
| MIPI |  |  |  |
| Low | 40 (49.4) | Reference | Reference |
| Intermediate | 23 (28.4) | 0.64, 0.21 – 2.00, p= 0.41 | 0.60, 0.22 – 1.63, p= 0.28 |
| High | 18 (22.2) | 1.04, 0.41 – 2.64, p= 0.93 | 0.89, 0.38 – 2.07, p= 0.78 |
| *TP53* mutation/ aberration |  |  |  |
| No mutation | 49 (73.1) | Reference | Reference |
| Mutation | 18 (26.9) | 1.48, 0.62 – 3.51, p= 0.32 | 1.34, 0.58 – 3.09, p= 0.46 |
| Ki-67 |  |  |  |
| 10-30% | 14 (20.1) | Reference | Reference |
| ≥30% | 53 (79.1) | 1.25, 0.54 – 2.89, p= 0.63 | 1.31, 0.57 – 2.99, p= 0.55 |
| Prior Auto-HSCT |  |  |  |
| No | 50 (44.2) | Reference | Reference |
| Yes | 63 (55.8) | 1.27, 0.69 – 2.34, p= 0.45 | 1.33, 0.77 – 2.30, p= 0.32 |
| Prior Allo-HSCT |  |  |  |
| No | 103 (91.2) | Reference | Reference |
| Yes | 10 (8.8) | 1.35, 0.41 – 4.33, p= 0.57 | 1.66, 0.58 – 4.72, p= 0.24 |
| Prior Bendamustine |  |  |  |
| No Bendamustine or Bendamustine > 1a prior CAR-T | 96 (86.5) | Reference | Reference |
| Bendamustine < 1a prior CAR-T | 13 (13.5) | 2.49, 0.85 – 7.32, p= 0.02 | 2.04, 0.76 – 5.52, p= 0.06 |
| POD12 |  |  |  |
| No | 40 (35.4) | Reference | Reference |
| Yes | 73 (64.6) | 2.06, 1.09 – 3.86, p= 0.05 | 1.40, 0.80 – 2.47, p= 0.26 |
| Pre-apheresis bridging |  |  |  |
| Response to bridging | 11 (9.7) | Reference | Reference |
| No bridging |  | 0.89, 0.30 - 2.66, p= 0.83 | 0.96, 0.38 – 2.43, p= 0.93 |
| No response to bridging |  | 2.07, 0.91 – 4.69, p= 0.06 | 2.19, 1.06 – 4.53, p= 0.02 |
| Type of pre-apheresis bridging |  |  |  |
| Venetoclax (mono or + CD20/RT) | 10 (9.9) | 0.23, 0.08 – 0.65, p= 0.11 | 0.35, 0.14 – 0.86, p= 0.12 |
| Chemotherapy | 35 (34.7) | 2.57, 1.25 – 5.29, p= 0.0015 | 2.3, 1.19 – 4.51, p= 0.0018 |
| BTKi (mono or + CD20/RT) | 28 (27.7) | 0.42, 0.20 – 0.89, p= 0.08 | 0.62, 0.32 – 1.18, p= 0.20 |
| BTKi + Venetoclax (+ CD20) | 11 (10.9) | 0.62, 0.24 – 1.6, p= 0.42 | 0.69, 0.29 – 1.66, p= 0.47 |
| Lenalidomide + CD20 | 11 (10.9) | 1.99, 0.64 – 6.20, p= 0.10 | 1.69, 0.64 – 4.50, p= 0.18 |
| Radiotherapy mono | 5 (5.0) | 0.49, 0.12 – 2.04, p= 0.47 | 0.81, 0.23 - 2.95, p= 0.77 |
| Anti-CD20 mono | 1 (1.0) | Not evaluated | Not evaluated |
| ZUMA-2 eligibility |  |  |  |
| No | 87 (77.0) | Reference | Reference |
| Yes | 26 (23.0) | 0.76, 0.36 – 1.59, p= 0.42 | 0.81, 0.42 – 1.58, p= 0.51 |
| Infections after CAR-T |  |  |  |
| No | 78 (69.0) | Reference | Reference |
| Yes | 35 (31.0) | 1.04, 0.54 – 2.03, p= 0.90 | 1.15, 0.61 – 2.19, p= 0.20 |
| IVIG substitution |  |  |  |
| No | 63 (57.8) | Reference | Reference |
| Yes | 46 (42.2) | 0.49, 0.26 – 0.90, p= 0.03 | 0.49, 0.28 – 0.87, p= 0.02 |

**Supplement 7**: Univariable analysis of patient factors and association with outcome.

Abbreviation: BTKI= Bruton’s tyrosine kinase inhibitor, CI= confidence interval, EFS= event-free survival, HR= hazard ratio, HSCT= hematopoetic stem cell transplantation, IVIG= intravenous immunoglobuline, OS = overall survival, POD12= Progression of disease within 12 months, RT= Radiotherapy

**Figure S8: OS according to patient baseline characteristics**

| A  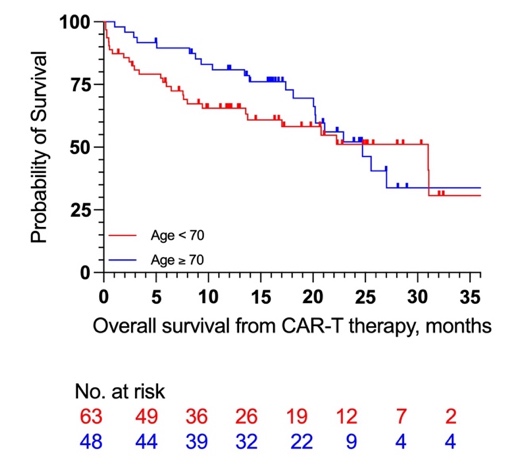 | B  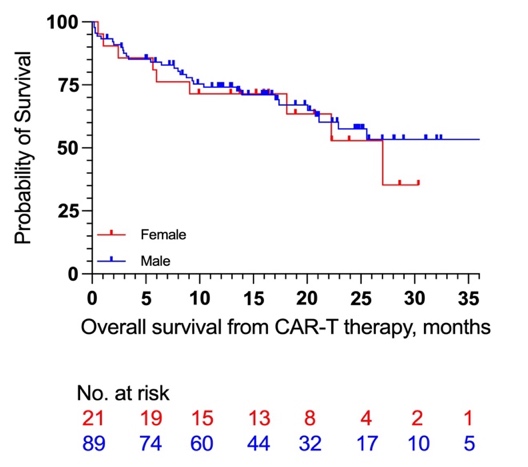 |
| --- | --- |
| C  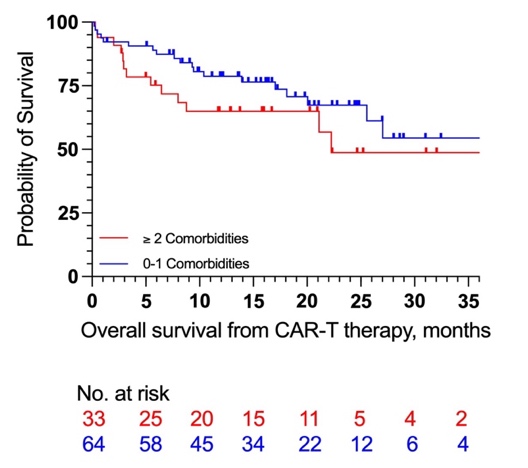 | D  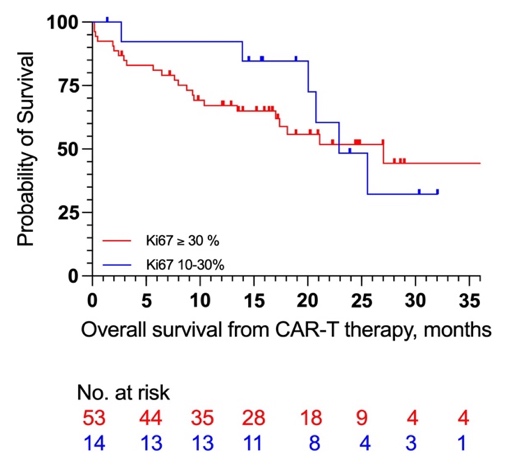 |
| E  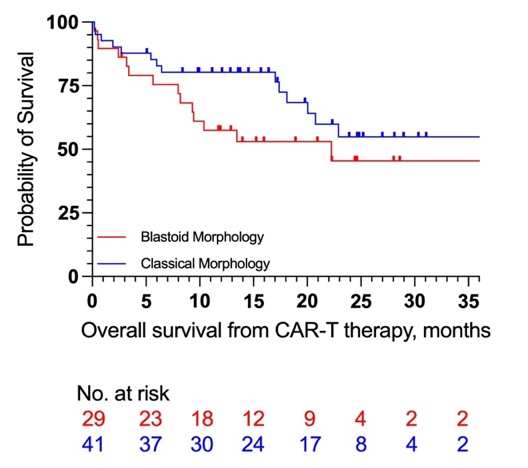 | F  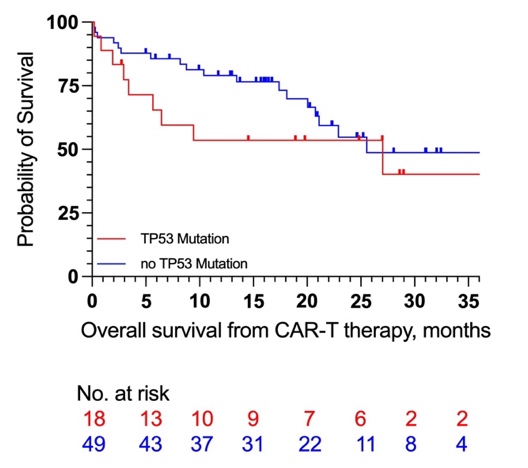 |
| G  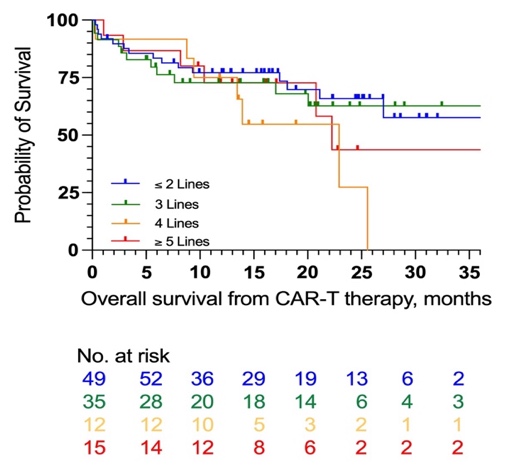 | H  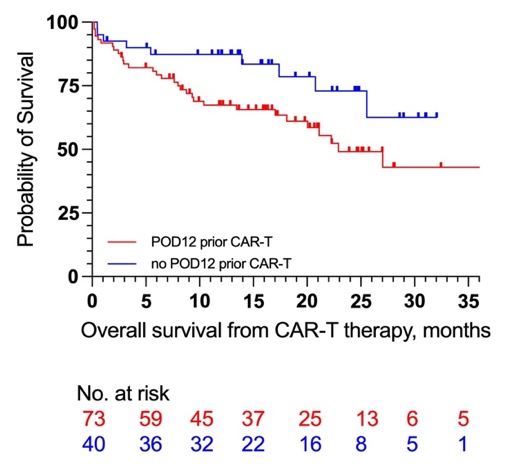 |
| I  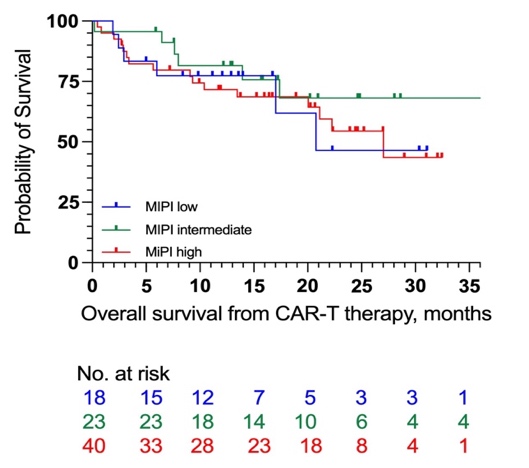 |  |

**Supplement 8**: OS stratified by patient characteristics. OS stratified by age (< 70 vs. ≥70) at CAR-T therapy (A), OS stratified by sex (B), OS stratified by number of comorbidities (C), OS stratified by Ki-67 index (<10-30 % vs. ≥ 30%) (D), OS stratified by morphology (classical vs. blastoid) (E), OS stratified by *TP53* mutations (F), OS stratified by lines of prior therapy (G), OS stratified by POD12 (H), OS stratified by MIPI (low vs. intermediate vs. high) (I).

**Figure S9: EFS according to patient baseline characteristics**

| A  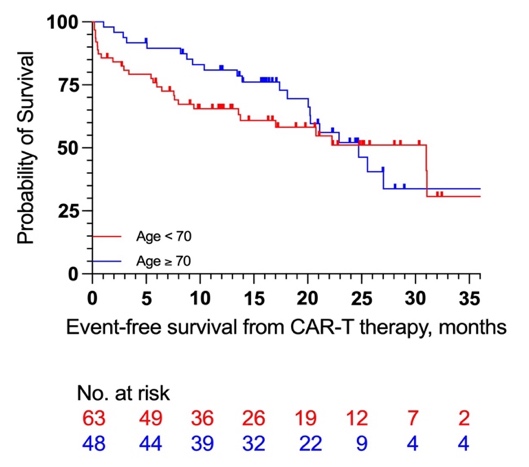 | B  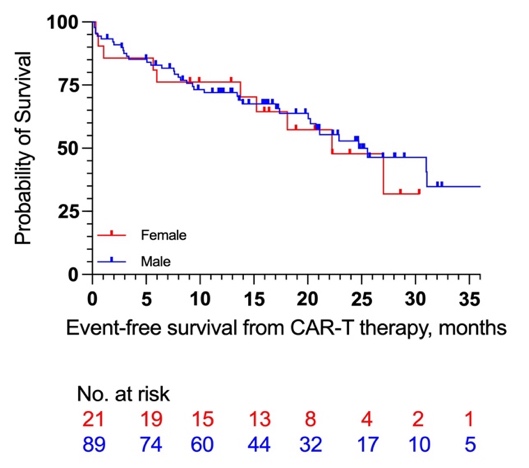 |
| --- | --- |
| C  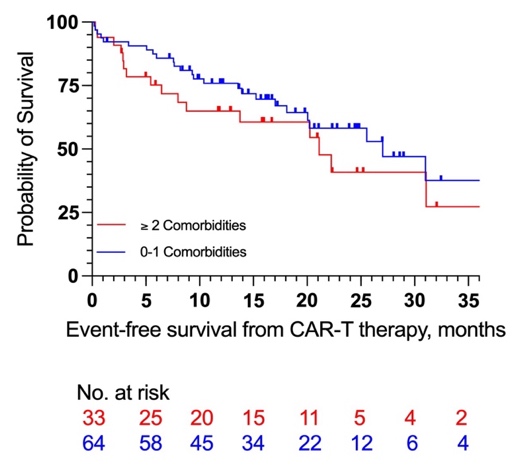 | D  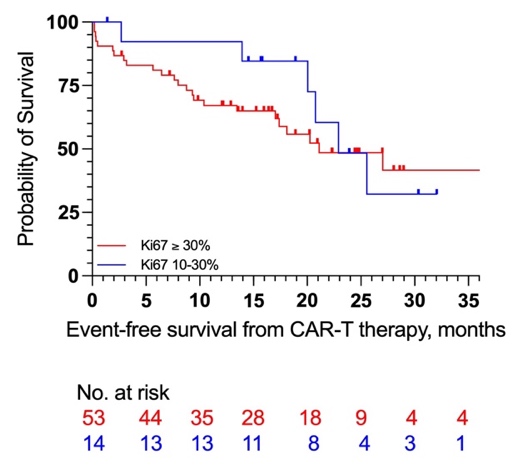 |
| E  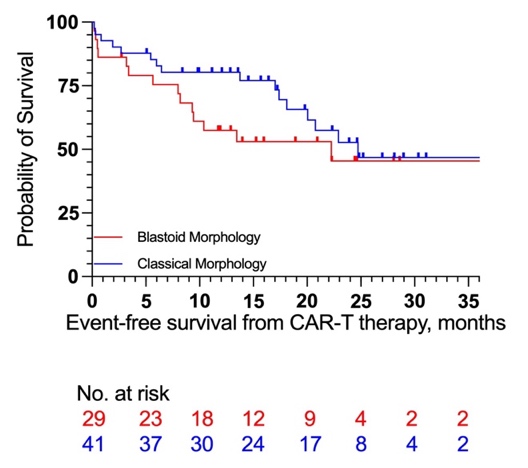 | F  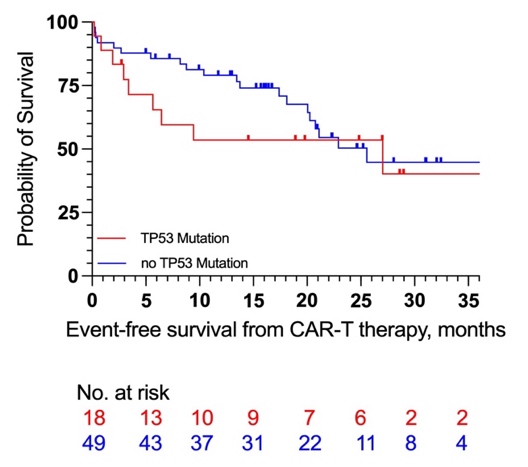 |
| G  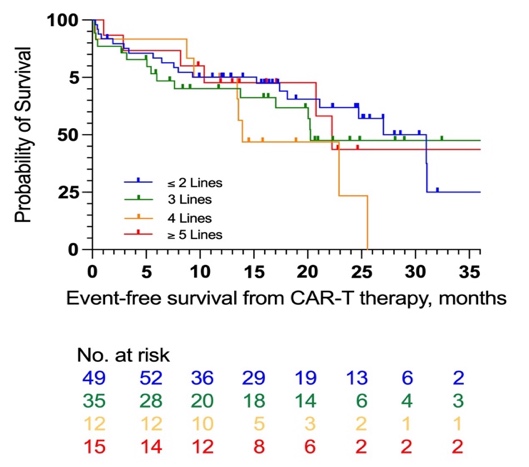 | H  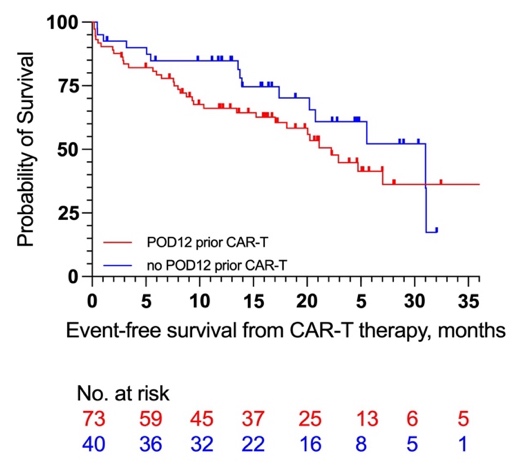 |
| I  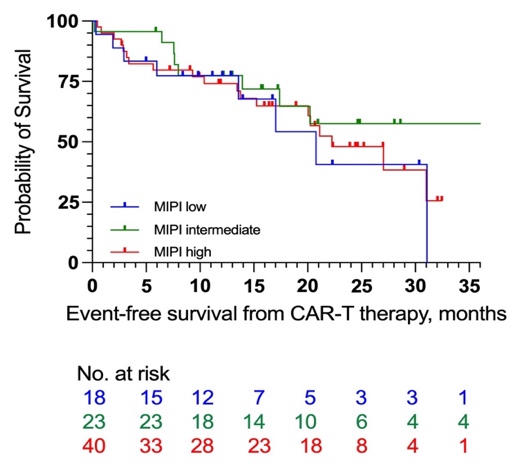 |  |

**Supplement 9**: EFS stratified by patient characteristics. EFS stratified by age (< 70 vs. ≥70) at CAR-T therapy (A), EFS stratified by sex (B), EFS stratified by number of comorbidities (C), EFS stratified by Ki-67 index (<10-30 % vs. ≥ 30%) (D), EFS stratified by morphology (classical vs. blastoid) (E), EFS stratified by *TP53* mutations (F), EFS stratified by lines of prior therapy (G), EFS stratified by POD12 (H), EFS stratified by MIPI (low vs. intermediate vs. high) (I).

**Figure S10: Survival stratified according to bridging**

| A  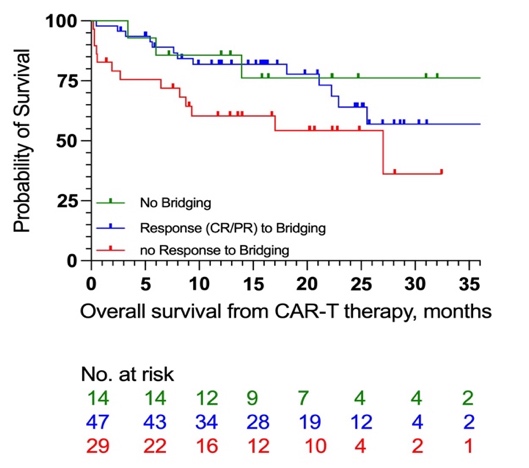 | B  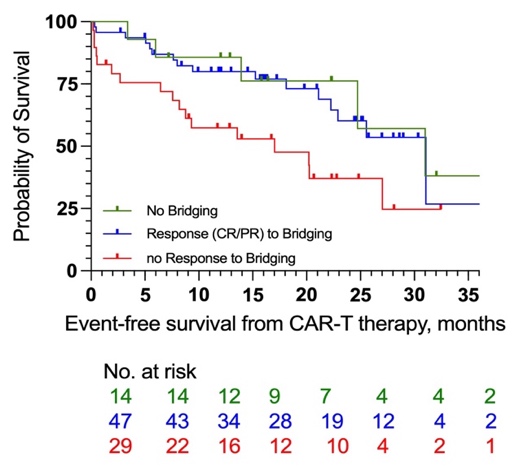 |
| --- | --- |
| C  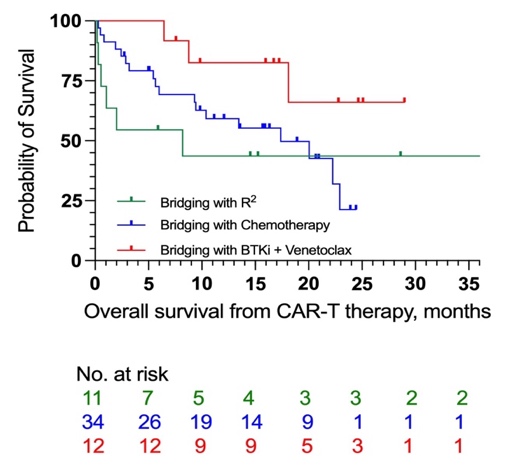 | D  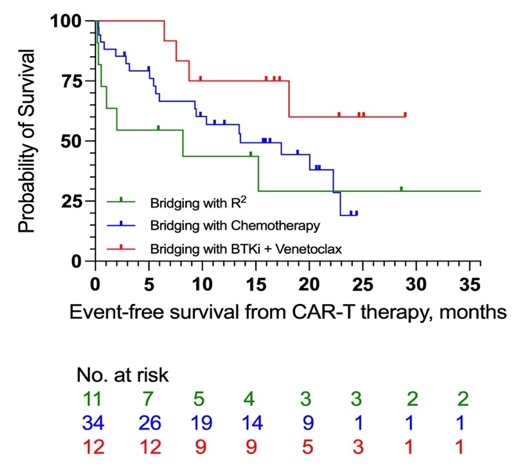 |

**Supplement 10**: Kaplan-Meier plots in regard to bridging strategies. (A) OS stratified by OR (CR/PR) vs. no OR (SD/PD) after bridging therapy, (B) EFS stratified by OR (CR/PR) vs. no OR (SD/PD) after bridging therapy, (C) OS stratified by most commonly used bridging strategies, and (D) EFS stratified by most used bridging strategies

**Table S11: Multivariable analysis of subgroups for overall survival**

| Variable | Standard Error | Wald-Chi-Quadrat | Pr > Chi² | Hazard Ratio | Hazard Ratio Lower Limit (95%) | Hazard Ratio Upper Limit (95%) |
| --- | --- | --- | --- | --- | --- | --- |
| Age, continous | 0.037 | 0.185 | 0.667 | 1.016 | 0.944 | 1.094 |
| Male sex | 0.713 | 0.168 | 0.682 | 1.339 | 0.331 | 5.422 |
| > 2 comorbidities | 0.532 | 2.419 | 0.120 | 2.286 | 0.806 | 6.481 |
| MIPI high | 0.769 | 1.940 | 0.164 | 0.342 | 0.076 | 1.547 |
| Zuma-eligible | 0.619 | 1.029 | 0.310 | 0.534 | 0.159 | 1.796 |
| POD12 | 0.657 | 5.883 | 0.015 | 4.919 | 1.358 | 17.823 |
| Prior HSCT | 0.649 | 0.095 | 0.757 | 0.818 | 0.229 | 2.922 |
| Ki67 > 30% | 0.649 | 2.294 | 0.130 | 0.374 | 0.105 | 1.335 |
| no bridging | 1.247 | 1.536 | 0.215 | 0.213 | 0.019 | 2.457 |
| no response to bridging | 0.583 | 1.249 | 0.264 | 1.919 | 0.612 | 6.018 |

**Supplement 11**: Multivariable analysis for OS.

Abbreviations: MIPI= Mantle-Cell-Lymphoma International Prognostic Index, POD12= progression of disease within 12 months, HSCT= hematopoetic stem cell transplantation

**Table S12: Multivariable analysis of subgroups for event-free survival**

| Variable | Standard Error | Wald-Chi-Quadrat | Pr > Chi² | Hazard Ratio | Hazard Ratio Lower Limit (95%) | Hazard Ratio Upper Limit (95%) |
| --- | --- | --- | --- | --- | --- | --- |
| Age, continous | 0.035 | 0.020 | 0.887 | 1.005 | 0.938 | 1.077 |
| Male sex | 0.711 | 0.210 | 0.647 | 1.385 | 0.344 | 5.579 |
| > 2 comorbidities | 0.516 | 2.762 | 0.097 | 2.356 | 0.858 | 6.473 |
| MIPI high | 0.726 | 1.588 | 0.208 | 0.401 | 0.097 | 1.662 |
| ZUMA-2-eligible | 0.578 | 1.850 | 0.174 | 0.455 | 0.147 | 1.415 |
| POD12 | 0.609 | 5.727 | 0.017 | 4.290 | 1.302 | 14.139 |
| Prior HSCT | 0.611 | 0.030 | 0.863 | 0.900 | 0.272 | 2.979 |
| Ki67 > 30% | 0.641 | 1.966 | 0.161 | 0.407 | 0.116 | 1.430 |
| no bridging | 1.204 | 1.241 | 0.265 | 0.261 | 0.025 | 2.770 |
| no response to bridging | 0.548 | 2.676 | 0.102 | 2.453 | 0.837 | 7.185 |

**Supplement 12**: Multivariable analysis for EFS.

Abbreviations: MIPI= Mantle-Cell-Lymphoma International Prognostic Index, POD12= progression of disease within 12 months, HSCT= hematopoetic stem cell transplantation

**Figure S13: Association of patient characteristics with OS (A) and EFS (B) depicted as forest plots**

| A  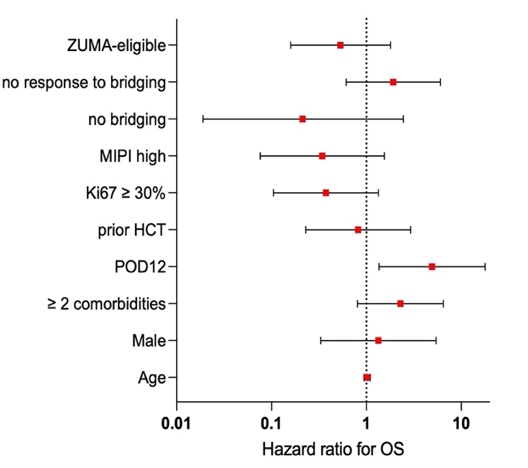 | B  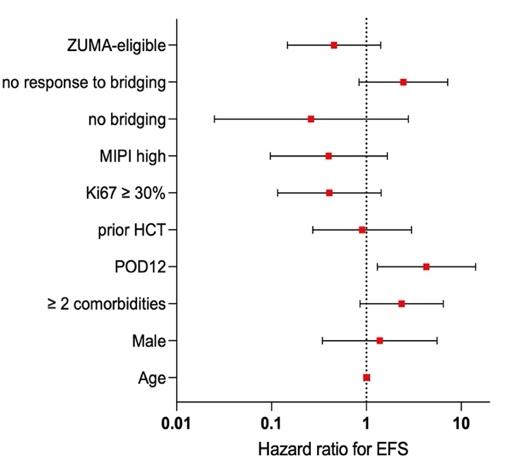 |
| --- | --- |

**Supplement 13**: Forest Plot for association of patient characteristics and OS (A) or EFS (B).

Abbreviations: MIPI= Mantle-Cell-Lymphoma International Prognostic Index, POD12= progression of disease within 12 months, HCT= hematopoetic stem cell transplantation

**Table S14: Toxicities and Infections during and post CAR-T therapy**

| (n=information available) | |  | All patients |
| --- | --- | --- | --- |
| CRS | total [n (%)] (n=109) | Yes  No | 93 (85.3)  16 (14.7) |
|  | Grade | 1  2  3  4  5 | 30 (32.3)  49 (52.7)  11 (11.8)  2 (2.2)  1 (1.1) |
| ICANS | total [n (%)] (n=107) | Yes  No | 58 (54.2)  49 (45.8) |
|  | Grade [n (%)] (n=107) | 1  2  3  4  5 | 22 (37.9)  20 (34.5)  7 (12.1)  9 (15.5)  0 (0) |
| ICU admission | total [n (%)] (n=103) | Yes  No | 23 (22.3)  80 (77.7) |
|  | Reason for ICU admission [n (%)] (n=23) | CRS  ICANS  Infection  other | 7 (30.4)  9 (39.1)  6 (26.1)  1 (4.3) |
|  | Duration of ICU stay, days [Median (range)] |  | 2 (1-10) |
| Infections and immune reconstitution post CAR-T therapy  (n=information available) | | | **All patients** |
| Infection after CAR-T therapy at any time | total [n (%)], (n=113) | Yes  No | 35 (31.0)  78 (69.0) |
| Type of infection, multiple options possible | total [n (%)], (n=67) | Bacterial Pneumonia  SARS-CoV-2-associated pneumonia  Other viral infection requiring treatment  Bacteremia with sepsis^1^  Urinary tract infection  Other^2^  unknown | 20 (29.9)  20 (29.9)  6 (9.0)  4 (6.0)  5 (7.5)  7 (10.4)  5 (7.5) |
| Recovery of immune cells 6 months post CAR-T therapy | B-cells [n (%)], (n=92)  T-cells [n (%)], (n=90) | Yes  No  Yes  No | 12 (13.0)  80 (87.0)  27 (30)  63 (70) |
| Substitution of Ig | total [n (%)] (n=109) | Yes  No | 46 (42.2)  63 (57.8) |

**Supplement 14**: ^1^ *Staphylococcus aureus* n=1, ESBL n=1, ^2^ meningitis n=1, toxoplasmosis n=1, CMV-reactivation n=2, *Clostridium difficile* infection n=1, fungal pneumonia n=2

Abbreviations: CMV = cytomegalovirus, CRS = Cytokine release syndrome, ESBL = extended spectrum beta-lactamase; ICANS = immune-effector associated neurotoxicity-syndrome, Ig = immunglobuline, SARS-CoV-2= Severe acute respiratory syndrome coronavirus, UTI = urinary tract infection

**Figure S15: OS and EFS according to occurrence of infection (A-B) and Ig substitution (C-F)**

| A  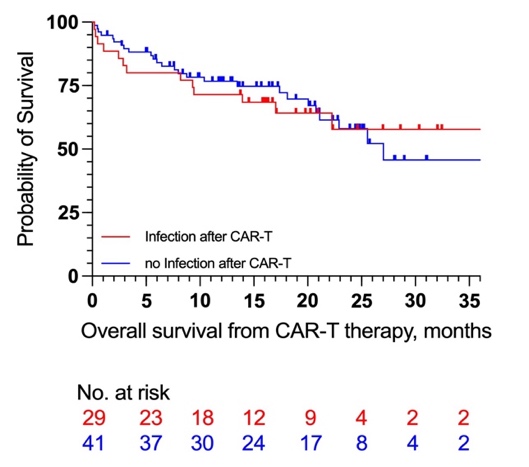 | B  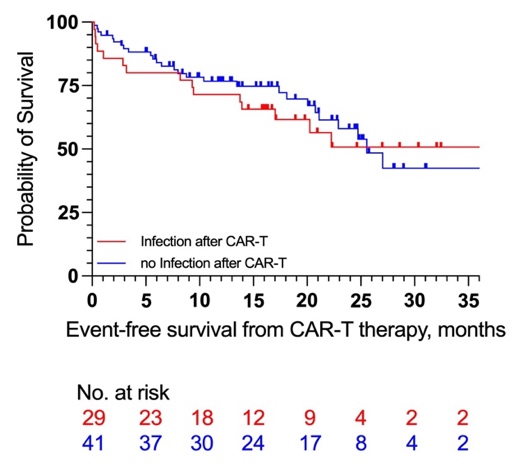 |
| --- | --- |
| C  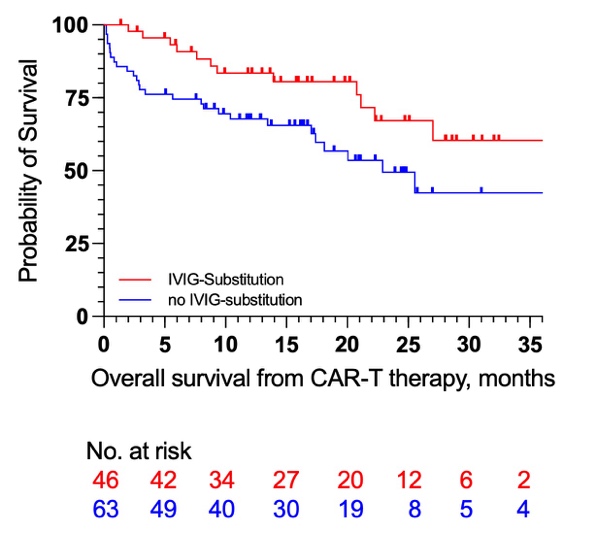 | D  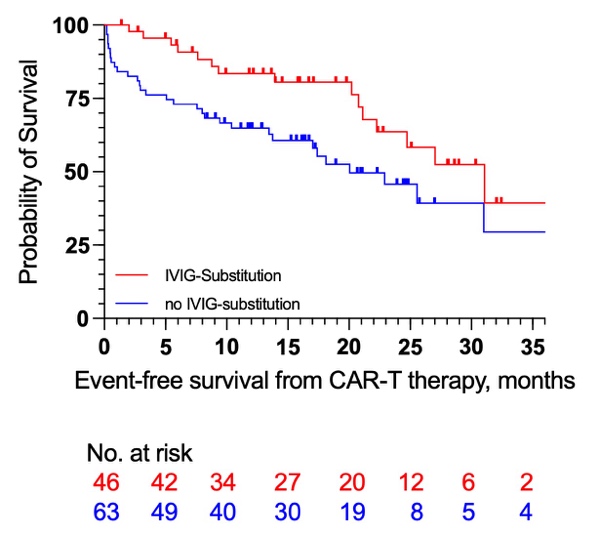 |
| E  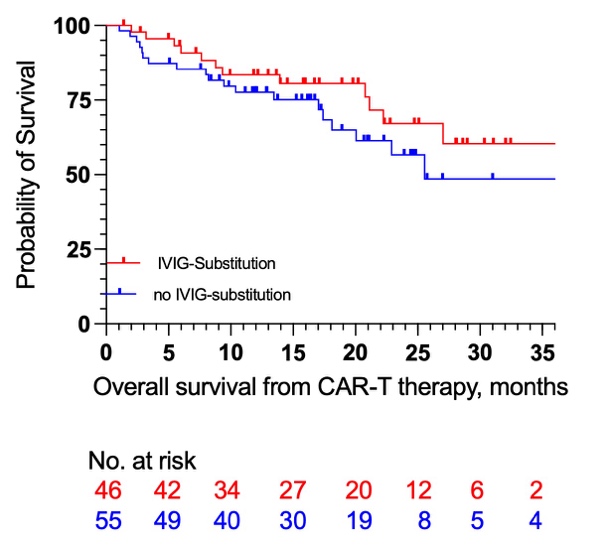 | F  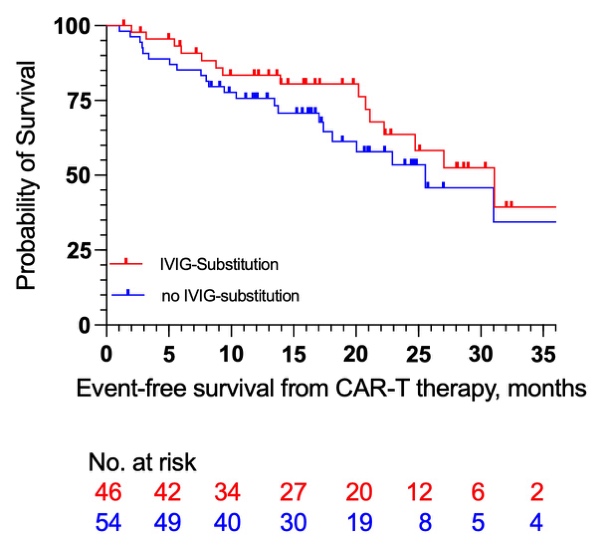 |

**Supplement 15:** OS (A) and EFS (B) for patients with or without significant clinical infection, OS (C) and EFS (D) for all patients with or without Ig-substitution, OS (E) and EFS (F) for patients with or without Ig-substitution with survival of at least 30 days after CAR-T administration.

**Table S16: Patient details for NRM**

| Days until death | Age at CAR-T, Sex | ECOG | Prior HSCT | Bridging | Refractory at LD | B-cell aplasia at death | Leading Cause of Death |
| --- | --- | --- | --- | --- | --- | --- | --- |
| 6 | M, 63 | 0 | auto | yes | yes | no | SARS-CoV-2 Pneumonia |
| 6 | M, 68 | 0 | auto | yes | n.a. | yes | refractory CRS V° with cardiogenic shock |
| 9 | M, 69 | n.a. | auto | yes | yes | yes | Renal insufficiency after CAR-T therapy, infection with respiratory distress |
| 9 | M, 65 | 3 | auto | yes | yes | yes | Cardiac failure with respiratory insufficiency and ICANS |
| 10 | F, 69 | n.a. | auto | yes | yes | unknown | CRS V with cardiogenic shock and tachyarrhythmia, use of cytokine adsorption |
| 15 | n.a., 64 | 1 | no | yes | yes | yes | ICANS IV, cardiogenic shock with tachyarrhythmica |
| 25 | M, 58 | 1 | no | yes | unknown | no | unknown |
| 32 | F, 70 | 1 | auto | yes | unknown | yes | Sepsis and MAS with MOV |
| 87 | M, 71 | 1 | auto | yes | unknown | yes | neutropenic infection (neutropenia, thrombopenia, anemia grade III°) |
| 88 | M, 62 | 0 | auto | yes | yes | yes | cardiogenic shock after lung embolism |
| 287 | M, 66 | 0 | auto, allo | yes | no | yes | Meningitis (*Elisabethkingia meningoseptica*) (neutropenia grade I°) |
| 424 | M, 78 | 1 | no | no | unknown | yes | Pneumonia, sepsis, multiple infections during long-term cytopenia (neutropenia, thrombopenia grade III°) |

**Supplement 16**: Details for patients with NRM as cause of death. Abbreviations: m=male, f=female, HSCT = hematopoietic cell transplantation, n.a. = not available, MAS = macrophage activation syndrome, MOV = multiorgan failure, CRS = cytokine release syndrome, ICANS = immune effector cell-associated neurotoxicity syndrome

**Table S17: Outcome after CAR-T-failure**

| (n=information available) |  | All patients evaluable |
| --- | --- | --- |
| Progression/Failure after CAR-T therapy [n (%)] (n=40) | Dead at End of Observation  Alive at End of Observation | 29 (72.5)  11 (27.5) |
| Median time from CAR-T therapy to MCL progression, months [Median (range)] |  | 13.6 (0.5-40.5) |
| OS after progression post CAR-T therapy | Median, months (range)  12 months [% (95% CI)] | 4.8 (0-33.8)  21.8 (8.6-38.8) |
| First post-CAR-T therapy [n (%)], (multiple options possible), (n=18) | Antibody + Chemotherapy^1^  Venetoclax  Lenalidomid  BTKi^2^  BTKi + Venetoclax  Other^3^ | 1 (5.6)  2 (11.1)  1 (5.6)  9 (50.4)  1(5.6)  4 (22.2) |
| Best response to first post-CAR-T therapy [n (%)] (n=18) | CR  PR  SD  PD  n.e.  unknown | 2 (11.1)  1 (5.6)  1 (5.6)  7 (38.9)  4 (22.2)  3 (16.7) |

**Supplement 17**: ^1^R-CHOP + methotrexate, ^2^Ibrutinib n=3, Nemtabrutinib n=2, Pirtobrutinib n=3, ^3^ allogeneic stem cell transplantation n=2, dexamethasone n=1, Epcoritamab n=1

Abbreviations: CR=complete remission, n.e. = not evaluated, n.r. = not reached, OS = overall survival, PD=progressive disease, PR=partial remission, SD=stable disease
